# Evaluation of Challenge Models for *Flavobacterium covae* Infection of Grass Carp (*Ctenopharyngodon idellus*)

**DOI:** 10.3390/microorganisms13102318

**Published:** 2025-10-07

**Authors:** Rui Han, Huicheng Wu, Zhongning He, Zequan Mo, Xueming Dan, Yanwei Li

**Affiliations:** 1 College of Marine Sciences, South China Agricultural University, Guangzhou 510642, China; cassierui@163.com (R.H.);; 2 Nansha-South China Agricultural University Fishery Research Institute, Guangzhou 511457, China

**Keywords:** *Flavobacterium covae*, infection model, immersion, intramuscular injection, intradermal injection

## Abstract

Columnaris disease is a highly contagious infection that affects nearly all freshwater fish species worldwide. Grass carp, one of the most economically significant freshwater fish species in China, is particularly susceptible to the disease, leading to large-scale mortality. *Flavobacterium columnare* and *F. covae* are the primary pathogens causing columnaris disease in Chinese grass carp aquaculture. Herein, we compare mortality rates, replication rates of typical columnaris symptoms, histopathological changes, and bacterial content in the tissues of grass carp following infection using four challenge models. The mortality rate in grass carp challenged via intraperitoneal injection was 86.7%. All fish infected via intramuscular and intradermal injections died, while immersion resulted in lower mortality. Gill corrosion rates were 67%, 53%, and 87%, respectively, in the intramuscular injection, intradermal injection, and immersion groups. Correspondingly, skin ulceration rates were 75%, 91%, and 63%. However, surface symptoms in the intraperitoneal injection group were milder. Histopathological analysis revealed similar lesions in grass carp subjected to immersion, intramuscular, and intradermal infection, which differed from carp infected via intraperitoneal injection. The trends in bacterial loads in the gills and skin were similar, although the absolute bacterial content varied between tissues. Bacterial loads in the immersion and intraperitoneal injection groups were lower than those in the other groups. Based on these findings, we determined that the optimal model for simulating columnaris disease in grass carp is the intradermal injection of *F. covae* in 10–12 cm fish. The infection model generated via intradermal injection resembles natural *F. covae* infection and can serve as a good tool for evaluating the protective effect of anti-*F. covae* infection vaccines in grass carp.

## 1. Introduction

Columnaris disease, named for the columnar aggregates formed by pathogenic bacteria, is an acute or chronic bacterial infection affecting freshwater fish. Clinically, columnaris disease is characterized by extensive skin lesions, fin erosion, and gill necrosis [1,2]. Lesions on the gills typically appear as gray or yellow necrotic areas with bacterial colonization. The skin ulcerates and turns white, with abscesses forming around the lesion, and forms a characteristic “saddleback” lesion [3]. This disease affects almost all freshwater fish species worldwide, including grass carp, channel catfish, salmonids, black mollies, eels, goldfish, perch, tilapia, and others [2,4,5,6,7,8]. It spreads rapidly and is associated with high mortality rates, leading to significant economic losses in both natural fish populations and the aquaculture industry worldwide [3,9,10]

The causative agent of columnaris disease was once thought to be *Flavobacterium columnare*, a Gram-negative, gliding bacterium that forms colonies with variable morphology. These bacteria exhibit four distinct colony morphologies on Shieh agar plates: rhizoid and flat, non-rhizoid and hard, round and soft, and irregularly shaped and soft [11,12,13]. Due to its genetic and morphological diversity, recent studies have reclassified *F. columnare* into four species: *F. columnare*, *F. covae* sp. nov., *F. davisii* sp. nov., and *F. oreochromis* sp. nov. [14]. Establishing reproducible challenge models is crucial for studying pathogenic microorganisms. These models simulate natural disease conditions in animals, which is essential for developing vaccines and other therapeutic interventions, as they cannot be fully replicated by cell cultures or computer simulations alone [15,16]. Research on columnaris disease challenge models has involved methods such as immersion baths, intraperitoneal injections, and intramuscular injections, although these approaches have limitations. Immersion infection, with or without abrasion, has been reported in species such as zebrafish and rainbow trout [4,17,18,19]. These studies often used small fish fry and small water volumes. Intraperitoneal injection of *F. columnare* resulted in a low mortality rate (~7%) in *Ictalurus punctatus*, and typical ulcerative symptoms on the gills and skin were not easily induced under natural infection conditions [20,21]. Although intramuscular injection of *F. columnare* can cause high mortality (~90%) in fish, the occurrence of typical natural symptoms post-infection remains unclear [4,18,20,21]. Currently, a standardized challenge model for columnaris disease-related research has yet to be established. Additionally, intradermal injection models have been used in mammalian studies, such as in female Sprague Dawley rats for *Propionibacterium acnes* infections and in murine models for visceral leishmaniasis [22,23]. Intradermal infection of rhesus macaques with Mpox resulted in numerous skin lesions and high plasma viral loads [24]. A new phagocytosis model was developed using intradermal methylene blue-labeled *Escherichia coli* injection, and a challenge model was established utilizing the attenuated vaccine agent *Mycobacterium bovis* BCG as a surrogate for *Mycobacterium tuberculosis*, with intradermal (skin) challenge as an alternative to pulmonary infection [25,26].

While intradermal injections are common in mammalian models, their application in fish remains relatively rare. Considering that previous studies have documented the accumulation of bacterial aggregates within collagen fiber networks of the fish dermis, we also tested intradermal injection as a challenge method.

Grass carp is the most significant farmed freshwater fish in China, with annual production exceeding 5 million tons. Columnaris disease is one of the most severe bacterial diseases affecting grass carp. Epidemiological surveys across various Chinese provinces have identified *F. columnare* as the main pathogen, followed by *F. covae*. The inactivated vaccine developed by our laboratory using the *F. covae* MU-04 strain provides effective immune protection against both *F. columnare* and *F. covae*. However, research on *F. covae* remains limited. A unified and reliable challenge model is crucial for future experiments to advance our understanding of *F. covae*’s pathogenic mechanisms and the host’s antibacterial defense, as well as to scientifically assess vaccine efficacy.

Building on previous studies, this research infected grass carp with the *F. covae* strain MU-04. We compared four challenge methods by evaluating induced mortality, the development of typical columnaris disease symptoms, pathological changes, and tissue bacterial loads. This comparison revealed the optimal infection model, which will facilitate research into the resistance mechanisms of grass carp against *F. covae* and support the development of effective vaccines.

## 2. Materials and Methods

### 2.1. Bacteria, Fish, and Sampling

The *F. covae* isolate MU-04 was cultured on modified Shieh agar plates and in Shieh broth with shaking at 28 °C and 200 rpm. The bacterial culture was centrifuged at 6000 rpm for 7 min; the supernatant was discarded, and the bacterial pellet was resuspended in PBS. The resuspended bacteria were serially diluted, and a plate count was performed to determine the bacterial concentration. Grass carp of various sizes (3–4 cm, 7–8 cm, 10–12 cm, and 13–15 cm) were sourced from the Xinxing Seedling Farm in Yunfu City, Guangdong Province. Before the experiment, the fish were tested for pathogens during the temporary rearing stage, and no pathogens were detected.

After anesthetizing the fish with MS-222, gill and skin samples were collected from three fish at each of the following time points: 0.5 d, 1 d, 2 d, 3 d, 4 d, 5 d, and 6 d post-infection. For gill sample collection, the gill tissue was carefully separated, rinsed thoroughly with sterile PBS three times to ensure cleanliness, and trimmed into appropriate sizes. For skin (including muscle) sample collection, a section of skin, along with the underlying muscle, was excised from the right side of the grass carp’s spine. The sample was then cut into suitable pieces and transferred to sterile Eppendorf tubes for further analysis. The collected gill and muscle tissue samples were divided into two parts: one part was fixed in 4% paraformaldehyde for paraffin sectioning and pathological examination; the other part was placed in a sterile microcentrifuge tube and stored at −80 °C for DNA extraction and quantification of bacterial load in the tissue.

### 2.2. Challenge Methods

The experimental fish were divided into five groups based on the infection method used: immersion, intraperitoneal injection, intramuscular injection, intradermal injection, and control groups. In the immersion group, the fish were further categorized based on the experimental objectives: (1) challenge experiments with grass carp of different sizes and (2) challenge experiments with varying bacterial concentrations. Each of these experiments included 3–4 subgroups.

#### 2.2.1. Immersion Group

For the size-based challenge experiment, grass carp were categorized according to size (3–4 cm, 7–8 cm, 10–12 cm, and 13–15 cm) and divided into four subgroups for the subsequent challenge. In each subgroup, 4 L, 6 L, 8 L, or 10 L of water was added, and MU-04 was introduced to achieve a final bacterial concentration of 1 × 10^7^ cfu/mL. After 4 h of immersion at 28 °C with aeration, the fish were removed, and surface bacteria were washed off with sterile water. The fish were then placed into a new 200 L barrel for normal breeding and observation.

For the immersion challenge, 10 cm grass carp were used with different bacterial concentrations. The fish were assigned to one of the four experimental groups based on bacterial concentration: high-infection group (1.3 × 10^10^ cfu/fish), medium-infection group (7 × 10^9^ cfu/fish), low-infection group (1.3 × 10^9^ cfu/fish), and a PBS immersion control group. In each of the experimental groups, 80 mL of the resuspended MU-04 bacterial solution was added to 8 L of water containing 15 grass carp. After 4 h of immersion at 28 °C with aeration, the fish were removed, washed in sterile water to eliminate surface bacteria, and placed into a 200 L barrel for normal breeding and observation.

#### 2.2.2. Intraperitoneal, Intramuscular, and Intradermal Injections

The three injection groups were further divided into three subgroups based on the bacterial concentration administered: high-infection group (5 × 10^8^ cfu/fish), medium-infection group (2.6 × 10^8^ cfu/fish), and low-infection group (5 × 10^7^ cfu/fish). A control group was injected with PBS. Each subgroup consisted of 15 grass carp, all measuring 10 cm in length.

For the intraperitoneal injection, 1 mL syringes were used to inject 0.1 mL of the resuspended bacterial solution into the abdominal cavity of each grass carp. In the intramuscular injection group, 0.1 mL of the resuspended bacterial solution was injected into the muscle along the left side of the grass carp’s spine. For intradermal injection, the syringe was gently inserted upward from the dorsal fin into the left side of the spine, and 0.1 mL of the bacterial solution was injected. Following the injection, a small bulge under the skin of the fish could be observed.

After challenge, the clinical symptoms and mortality of the experimental fish were monitored and recorded daily. The recorded symptoms included the accumulation of yellow sticky bacteria, gill ulceration, skin damage, and the appearance of “saddleback” lesions.

### 2.3. Tissue Sections and Hematoxylin–Eosin Staining 

First, the collected samples were fixed in paraformaldehyde for 24 h. After fixation, specimens were rinsed once with double-distilled water (ddH_2_O) and trimmed. Subsequently, a graded ethanol dehydration series was performed: samples were immersed sequentially in 70%, 80%, and 90% ethanol for 40 min each, followed by two 20-minute incubations in absolute ethanol (with solution replacement after the first cycle). Tissues were then treated with a 1:1 ethanol–xylene mixture for 30 min. Following mixture removal, pure xylene was added to achieve full tissue transparency. The transparent tissues were transferred to a pre-warmed paraffin bath at 65 °C for 2 h of immersion. Finally, tissues were embedded in paraffin blocks. After cooling, slices were placed in a 46 °C water bath, spread and attached to adhesive slides, and put in a 60 °C oven for 4 h until the tissue slice was completely attached to the slide.

Tissue sections were dewaxed by immersing them in xylene for 7 min, repeated once. The sections were then transferred to a 1:1 ethanol–xylene mixture for 5 min. Subsequent rehydration was performed through a graded ethanol series: 100%, 90%, 80%, and 70% ethanol (3 min each), followed by a 3-minute rinse under water. Nuclei were stained with hematoxylin for 6 min (duration adjustable based on staining intensity) and then rinsed under water for 3 min. Differentiation was achieved using 0.5% acid ethanol, after which sections were rinsed with water and returned to blue in 1% ammonia, followed by a final water rinse. Cytoplasmic staining was performed with eosin solution for 2 min. Sections were then dehydrated through immersion in 95% ethanol twice (5 min each) and 100% ethanol twice (5 min each) and finally cleared in xylene twice (5 min each). After their removal from xylene, the sections were mounted with neutral balsam.

### 2.4. Establishment of qPCR Detection Method

#### 2.4.1. Plasmid Standard Preparation and Standard Curve Construction

Genomic DNA from MU-04 was extracted using the OMEGA Bacterial DNA Kit (Omega Bio-Tek, Inc., Norcross, GA, USA), according to the manufacturer’s instructions. The gyrB gene sequence was identified within the MU-04 genome, and SYBR primers (qgyrB-F: 5′-ATTTCTGGTGACGACTTC-3′; qgyrB-R: 5′-CTACTGCTTGGGATACTG-3′) were designed using Beacon Designer software (8.14). The amplification was carried out using the following PCR protocol: A 50 μL reaction mixture was prepared, containing 1 μL of MU-04 genomic DNA template, 25 μL of PrimeSTAR Max Premix (TAKARA, Bio, Shiga, Japan), 2 μL of forward primer (qgyrB-F), 2 μL of reverse primer (qgyrB-R), and 20 μL of distilled water. The PCR conditions included an initial denaturation at 98 °C for 10 s, followed by 34 cycles at 60 °C for 15 s, 72 °C for 90 s, and a final extension at 72 °C for 5 min.

The target gel band was extracted and purified using the OMEGA Gel Extraction Kit, ligated into the pMD 18-T vector, and incubated at 16 °C for 3 h. The recombinant plasmid was then transformed into *E. coli* DH5α competent cells. Following overnight incubation at 37 °C, single colonies were selected and identified using PCR with the universal M13F/R primers. The PCR products of the correct size were sequenced by BGI, and positive colonies were cultured for further analysis. The gyrB plasmid was extracted using the OMEGA Plasmid Mini Kit I (Omega Bio-Tek, Inc.), and the plasmid concentration was determined.

The copy number of gyrB in the 1 μL plasmid standard solution was calculated using the following formula: X = (Plasmid concentration (g/μL) × 6.02 × 10^23^)/(Molecular weight of recombinant plasmid × 660). The plasmid was then serially diluted in a 10-fold concentration gradient and used as a template for qPCR. The qPCR system consisted of 1 μL of plasmid template, 5 μL of SYBR Green Realtime PCR Master Mix (TOYOBO, Osaka, Japan), 0.3 μL of forward primer (qgyrB-F), 0.3 μL of reverse primer (qgyrB-R), and 3.4 μL of distilled water. The qPCR conditions were as follows: initial denaturation at 95 °C for 60 s, followed by 40 cycles of 95 °C for 15 s, 60 °C for 15 s, and 72 °C for 45 s. A melt curve analysis was performed at 95 °C for 15 s, 60 °C for 15 s, 95 °C for 15 s, and 33 °C for 15 s. A standard curve was generated by plotting the qPCR Ct values (*Y*-axis) against the plasmid concentrations (*X*-axis), and the formula was derived using Excel.

#### 2.4.2. qPCR Specificity and Sensitivity Detection and Method Application

Several pathogenic bacterial strains have been previously isolated from grass carp in our laboratory, including *Chryseobacterium* sp., *Flavobacterium indicum*, *Novosphingobium panipatense*, *Aeromonas veronii*, *Stenotrophomonas* sp., and *Aeromonas hydrophila*. The genomic DNA of these strains was extracted and used as the template for the qPCR assay according to the procedure described in Section 2.4.1.

For sensitivity detection, the genomic DNA solution was diluted according to a 10-fold concentration gradient and used as the new template. The qPCR products were verified through nucleic acid electrophoresis.

Gill samples from both diseased and healthy grass carp were collected, weighed, and recorded separately. To each sample, 1 mL of TE buffer and steel beads were added, and the gills were homogenized. Bacterial DNA from both diseased and healthy gills was extracted following the protocol of the OMEGA Bacterial DNA Kit (Omega Bio-Tek, Inc., Norcross, GA, USA). These DNA samples were then used as templates for qPCR and PCR detection, as outlined in a previous study [27].

### 2.5. Detection of Bacterial Loads in Grass Carp Tissues

The weights of the grass carp gills and skin samples collected previously (Section 2.1) were recorded. Following this, 1 mL of TE buffer and steel beads were added to each sample, and a tissue grinder was used to homogenize the tissues. The bacterial DNA from the different tissues was extracted according to the instructions provided by the OMEGA Bacterial DNA Kit (Omega Bio-Tek, Inc.). To quantify bacterial loads in the tissues, the extracted bacterial DNA was used as a template in qPCR assays. The corresponding Ct values at various time points post-challenge were used to generate a standard curve, allowing the calculation of bacterial loads, which were then normalized by tissue weight to determine the bacterial copy number (per mg of tissue).

### 2.6. Statistical Analysis

Significance analysis was performed using the *t*-test and one-way analysis of variance in SPSS 20 and GraphPad Prism 8 software. Data are presented as the mean ± standard deviation (SD), and graphs were generated accordingly.

## 3. Results

### 3.1. Challenge Experiment

#### 3.1.1. Immersion Challenge of Different Sizes of Grass Carp

**Figure 1:**
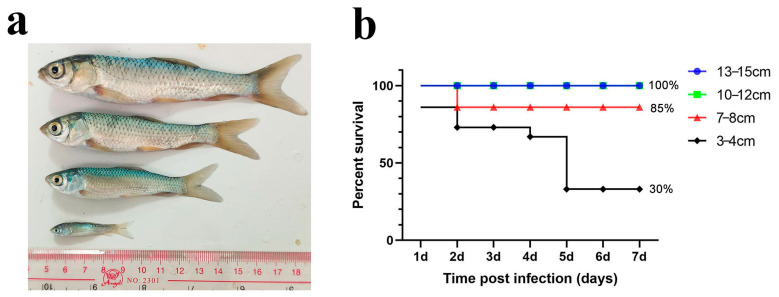
Immersion challenge of grass carp in different sizes. (**a**) Schematic diagram of grass carp in different sizes; (**b**) percent survival curve of grass carp after the immersion challenge.

We first performed an immersion challenge on grass carp of four different sizes (3–4 cm, 7–8 cm, 10–12 cm, and 13–15 cm) with a final bacterial concentration of 1 × 10^7^ cfu/mL (Figure 1a). After several trials, we found that grass carp in the 13–15 cm and 10–12 cm groups did not die during the experiment. The survival rate of the 7–8 cm group was 85%, while that of the 3–4 cm group was 30%. These results indicate that the immersion challenge caused higher mortality in smaller grass carp but did not increase mortality in larger individuals. Additionally, the typical columnaris symptoms were difficult to replicate in larger fish (Figure 1b).

#### 3.1.2. Different Challenge Methods for Grass Carp of the Same Size

These findings suggest that the immersion method does not result in high mortality. Therefore, we explored various injection methods to evaluate their effectiveness by comparing mortality and the incidence of typical columnaris symptoms in grass carp of the same size after challenge, with immersion serving as a control.

##### Immersion Challenge (Imm)

**Figure 2:**
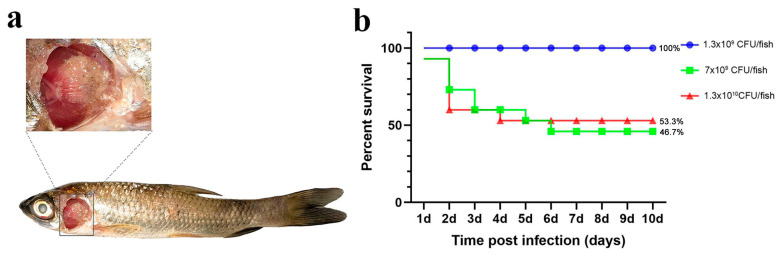
Immersion challenge. (**a**) Grass carp symptoms after the challenge; (**b**) percent survival curve of grass carp after the immersion challenge.

As a control, we applied three different challenge doses (high, medium, and low) and recorded the number of deaths and the symptoms in grass carp following the immersion challenge. In the highest-dose group, a significant number of deaths occurred within 1–2 days post-infection, with final relative survival rates of 53.3% (high dose), 46.7% (medium dose), and 100% (low dose; Figure 2).

While the three dose groups challenged via immersion did not die in large numbers, the proportion of dead fish exhibiting gill and skin symptoms was higher than in the other groups (Table 1). This suggests that the immersion method can simulate natural disease, but it does not guarantee high mortality, and the effect of the challenge is variable.

##### Intraperitoneal (IP) Injection

**Figure 3:**
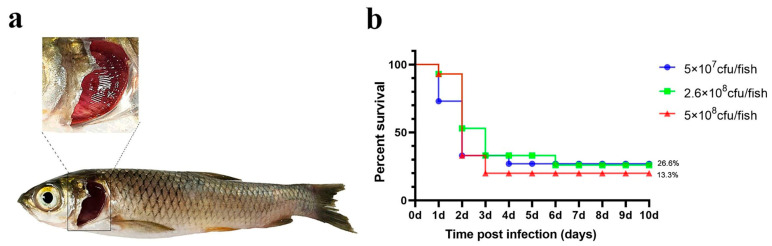
Intraperitoneal injection challenge. (**a**) Grass carp symptoms after the challenge; (**b**) percent survival curve of grass carp after the intraperitoneal injection challenge.

For the injection experiments, three bacterial doses (high: 5 × 10^8^ cfu/fish; medium: 2.6 × 10^8^ cfu/fish; low: 5 × 10^7^ cfu/fish) were used. The number of deaths and symptoms (including gill and skin lesions) in grass carp were recorded. Figure 3 shows that a significant number of fish died within 1–2 days of challenge, with final relative survival rates of 13.3% (high dose), 26.6% (medium dose), and 26.6% (low dose).

Although a significant number of grass carp died following intraperitoneal injection challenge, the replication rate of typical columnaris disease symptoms was low, and gill symptoms were not pronounced (Table 1).

##### Intramuscular (IM) Injection

**Figure 4:**
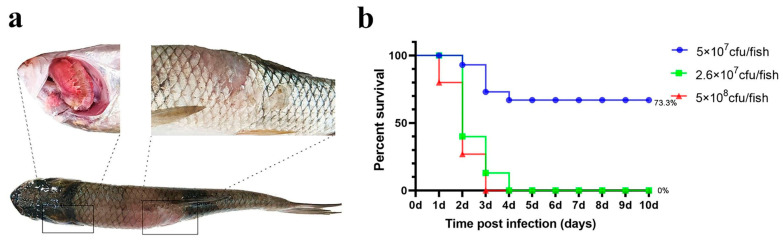
Intramuscular injection challenge. (**a**) Grass carp symptoms after challenge; (**b**) percent survival curve of grass carp after the intramuscular injection challenge.

The results are illustrated in Figure 4. In both the high- and medium-dose infection groups, the grass carp survival rates were 0%, whereas the survival rate in the low-dose group was 73.3%.

Notably, ulcerative lesions were observed on the dorsal muscle, and yellow erosive areas were clearly visible on the gills. The probability of typical symptoms appearing in deceased fish was high (Table 1).

##### Intradermal (ID) Injection

**Figure 5:**
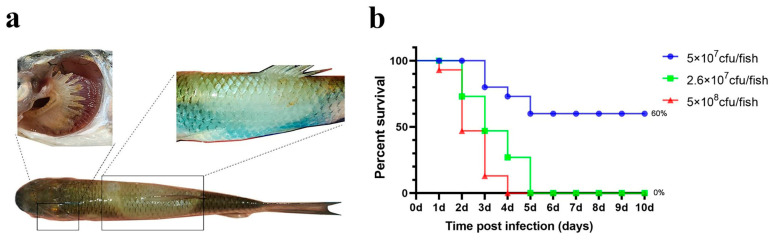
Intradermal injection challenge. (**a**) Grass carp symptoms after the challenge; (**b**) percent survival curve of grass carp after the intradermal injection challenge.

For the same bacterial dose, the survival rate of grass carp in the high- and medium-dose groups remained 0%, while the survival rate in the low-dose group dropped to 60% (Figure 5).

Grass carp exhibited distinctive “saddleback” lesions on the dorsal fin. Additionally, some deceased fish showed yellow biofilm around the edges of the lesion, and their gills were noticeably filled with sticky yellow substances (Figure 5). The rate of typical symptom development was similarly high (Table 1).

According to the results in this section, the mortality and symptom replication rate in grass carp differed following the four distinct infection methods. Intradermal injection of *F. covae* in grass carp resulted in high mortality and had a high probability of replicating typical columnaris symptoms.

### 3.2. Pathological Changes Induced by Different Challenge Methods

#### 3.2.1. Pathological Changes in Grass Carp Gills

**Figure 6:**
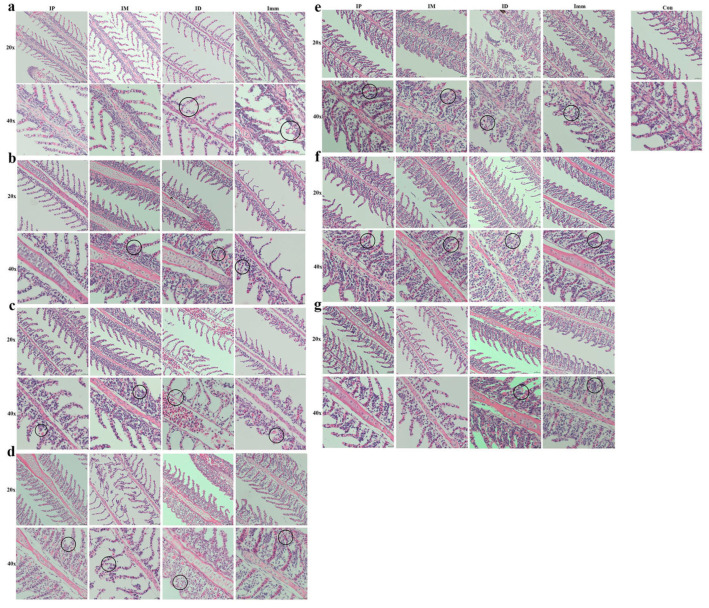
Pathological changes in grass carp gills after different challenge methods. IP: intraperitoneal injection; IM: intramuscular injection; ID: intradermal injection; Imm: immersion bath. The upper pictures were taken with a 20× objective lens, and the lower ones with a 40× objective. Images were taken (**a**) 12 h, (**b**) 1 d, (**c**) 2 d, (**d**) 3 d, (**e**) 4 d, (**f**) 5 d, and (**g**) 6 d post-challenge.

Immersion challenge (Imm): At 12 h post-infection, the grass carp gills in this group exhibited lamellar edema coincident with respiratory epithelial swelling (Figure 6a). By 1 dpi, persistent edema and epithelial hyperplasia were observed, accompanied by partial detachment of filament epithelium, and hyperplastic progression intensified through 2 dpi, peaking in severity at 3 dpi (Figure 6b–d). At 4–6 dpi, sustained epithelial hyperplasia remained the predominant histopathological feature, though with gradual subsidence of acute inflammatory changes (Figure 6e–g).

Intraperitoneal injection (IP): Within 1 dpi, grass carp in the intraperitoneal injection group exhibited no discernible pathological alterations (Figure 6a,b). At 2 dpi, lesions emerged in the gill filaments, primarily characterized by hyperplasia of the lamellar epithelium and basal cells, and this hyperplastic pattern persisted through 3–4 dpi (Figure 6c–e). The peak pathological manifestation occurred at 4 dpi, marked by maximum epithelial proliferation and tissue disorganization. Commencing at 5 dpi, lesion regression was observed, evidenced by reduced cellular hyperplasia and architectural restoration (Figure 6f,g).

Intramuscular injection (IM): Within 12 hpi, no significant pathological alterations were observed in the gills of the intramuscular injection group (Figure 6a). At 1–2 dpi, hyperplastic foci emerged in the basal epithelial cells of the gill lamellae (Figure 6b,c); by 3 dpi, cellular necrosis developed, with the sloughing of affected tissues (Figure 6d). At 4–5 dpi, progressive lamellar epithelial proliferation was evident and progressed to partial lamellar fusion. Improvement was observed at 6 dpi (Figure 6e–g).

Intradermal injection (ID): At 12 h post-infection, grass carp in the intradermal injection group exhibited mild edema and swelling of the gill epithelium, though less severe than in the immersion group (Figure 6a). By 2 dpi, slight hemorrhage and hyperplasia were observed within the gill lamellae (Figure 6b,c). At 3 dpi, hemorrhage intensified, accompanied by persistent epithelial swelling (Figure 6d). The most severe proliferative response occurred at 4 dpi, characterized by fusion of secondary lamellae in localized areas (Figure 6e). Subsequent days (5–7 dpi) showed sustained cellular hyperplasia at the lamellar base, albeit with marginal improvement (Figure 6f,g).

#### 3.2.2. Pathological Changes in Grass Carp Muscles

**Figure 7:**
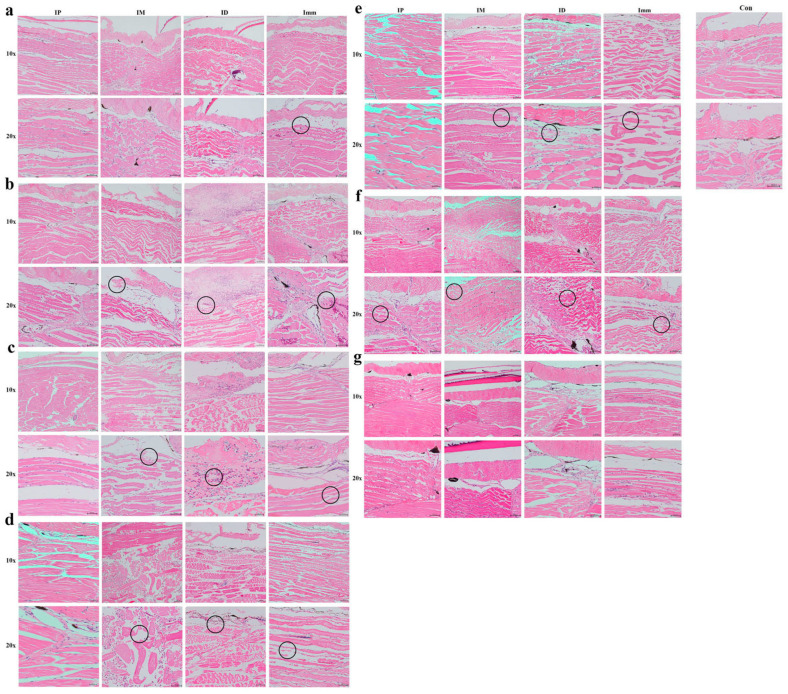
Pathological changes in grass carp skin (muscle) after different challenge methods. IP: intraperitoneal injection; IM: intramuscular injection; ID: intradermal injection; Imm: immersion bath. The upper pictures were taken with a 20× objective lens, and the lower with a 40× objective. Images were captured (**a**) 12 h, (**b**) 1 d, (**c**) 2 d, (**d**) 3 d, (**e**) 4 d, (**f**) 5 d, and (**g**) 6 d post-challenge.

Immersion challenge (Imm): At 12 hpi, the epidermal layer exfoliated in this group of grass carp, with slight tearing observed in the dermis. Minor petechial hemorrhages were present in the interstitial tissue between muscles, accompanied by an increase in inflammatory cells (Figure 7a). At 1 dpi, mild muscle atrophy occurred, with increased interstitial hemorrhage (Figure 7b). At 2–3 dpi, inflammatory cells continued to accumulate, accompanied by muscle atrophy and partial fiber rupture, with symptoms more severe than at 2 dpi (Figure 7c,d). At 4–5 dpi, muscle symptoms slightly improved, although atrophy persisted (Figure 7e,f). By 6 dpi, marked improvement was observed (Figure 7g).

Intraperitoneal injection (IP): In this group, the skin and muscle structures remained largely intact from 12 h to 3 days post-challenge, with no significant pathologies observed (Figure 7a,d). A slight elevation in inflammatory cell count was noted compared to the control group during this period. At 4–6 dpi, mild muscle atrophy emerged, accompanied by increased inflammatory cell infiltration (Figure 7e–g).

Intramuscular injection (IM): At 12 hpi, focal epidermal detachment was observed, accompanied by mild muscle atrophy and increased inflammatory cell infiltration, with no significant dermal changes (Figure 7a). At 1 dpi, partial dermal rupture and progressive muscle atrophy developed (Figure 7b); muscle fibers exhibited rupture, atrophy, and dissolution by 2 dpi (Figure 7c). At 3 dpi, pathological changes reached maximum intensity, characterized by muscle fiber atrophy, degeneration, necrosis, advanced myolysis, and inflammatory cell infiltration into the interstitial spaces (Figure 7d). Clear improvement was observed at 4–6 dpi compared to 3 dpi (Figure 7e–g).

Intradermal injection (ID): Grass carp in the intradermal injection group showed pathological manifestations comparable to those in the intramuscular injection group at 12 hpi, namely, mild muscular atrophy and elevated inflammatory cell infiltration (Figure 7a). By 1 dpi, extensive inflammatory cell infiltration was observed, alongside muscle fiber atrophy and degeneration, with focal myolysis (Figure 7b). The lesions at 2 dpi were characterized by advanced necrosis, hemorrhagic foci, and intensified inflammatory infiltration (Figure 7c). Progressive deterioration culminated at 3 dpi with sarcoplasmic coagulation and lysis, indicating widespread muscular necrosis (Figure 7d). Partial clinical improvement commenced at 4 dpi, followed by marked pathological regression by 6 dpi, where only localized inflammatory infiltration persisted (Figure 7e–g). This temporal progression highlights the characteristic myopathic cascade following experimental challenge.

This section describes the temporal histopathological changes in the gill and skin (muscle) tissues of grass carp under four distinct infection models. The three groups other than the intraperitoneal injection group showed edema and hyperplasia of gill lamellae, degeneration of muscle fibers, and focal myolysis.

### 3.3. Establishment of qPCR Method

#### 3.3.1. qPCR Standard Curve Construction

**Figure 8:**
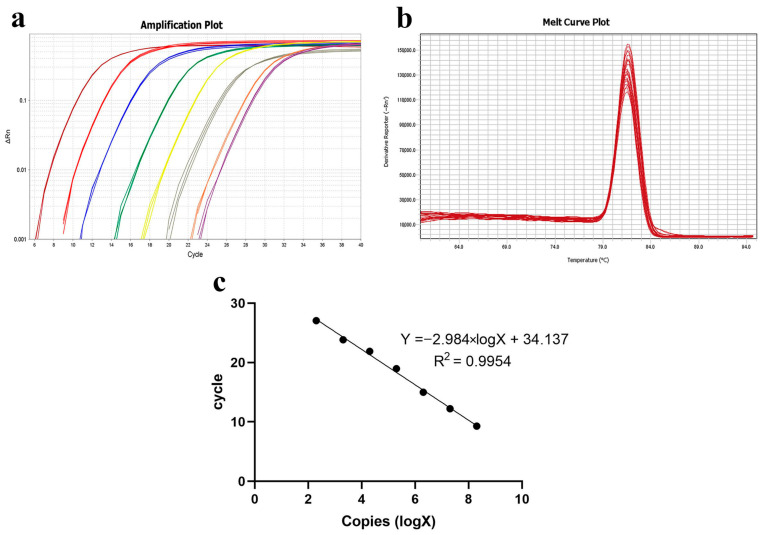
Establishment of qPCR standard curve. (**a**) Amplification curve plots of plasmid standards of different concentrations; (**b**) melt curve plots of plasmid standards of different concentrations; (**c**) standard curve of qPCR.

The vector plasmid solution was extracted and diluted to produce an eight-concentration gradient. The melting curves of the plasmid standards at varying concentrations demonstrated a linear relationship. The standard curve, with copy number as the *X*-axis and Ct value as the *Y*-axis, set the detection positivity threshold at Ct < 34.137 (Figure 8).

#### 3.3.2. Specificity of qPCR Detection

**Figure 9:**
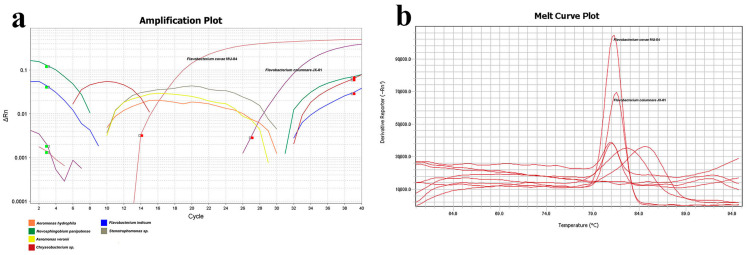
qPCR primer specificity detection. (**a**) Amplification plot of different strains. Orange line: *Aeromonas hydrophila*; green: *Novosphingobium panipatense*; yellow: *Aeromonas veronii*; red: *Chryseobacterium* sp.; blue: *Flavobacterium indicum*; gray: *Stenotrophomonas* sp. (**b**) Melt curve plot of different strains. *F. columnare* JX-01 and *F. covae* MU-04 have been marked above the curve.

To assess primer specificity, several bacteria previously isolated from the gills of grass carp were selected for qPCR detection of *F. columnare* JX-01 and *F. covae* MU-04 under identical conditions. The results revealed that only *F. covae* MU-04 generated detectable Ct values, with other bacteria showing undetermined Ct values, discontinuous amplification curves, and melting curve Tm values inconsistent with *F. covae* MU-04 (Figure 9, Table 2).

#### 3.3.3. Sensitivity of qPCR Detection

**Figure 10:**
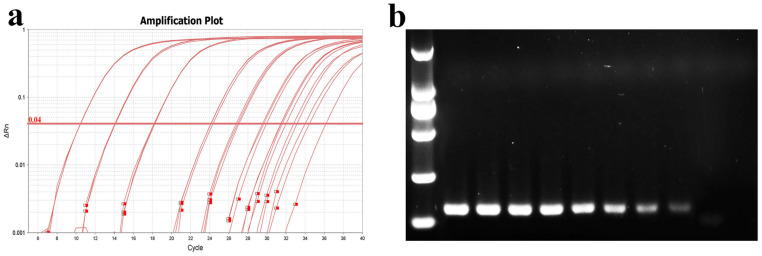
Determination of qPCR sensitivity. (**a**) Amplification curve plots of MU-04 gDNA at different concentrations; (**b**) nucleic acid gel electrophoresis of products after qPCR amplification. The template concentrations in (**a**,**b**) correspond to one another. The DNA concentrations from left to right in (**b**) are 375 ng, 37.5 ng, 3.75 ng, 0.375 ng, 37.5 pg, 3.75 pg, 0.375 pg, and 37.5 fg.

For sensitivity testing, a 10-fold dilution gradient of MU-04 gDNA was used as the template. The amplification protocol was as previously described, and qPCR products were subjected to nucleic acid electrophoresis. The results showed that the average Ct values for different gradient concentrations were 10.5, 14, 18, 24, 27, 30, 32, and 35. The PCR band intensity decreased with lower template concentrations, with sensitivity reaching 37.5 fg/10 µL reaction system (Figure 10).

#### 3.3.4. Application of qPCR Detection Method

**Figure 11:**
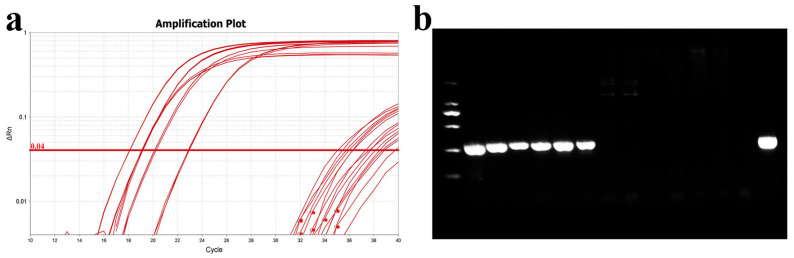
Comparison of qPCR and PCR assays. (**a**) Amplification curve plots of DNA from normal and diseased grass carp gills; (**b**) PCR detection of DNA from normal and diseased grass carp gills. From left to right are diseased grass carp and normal grass carp, and the last lane is the MU-04 gDNA positive control.

To validate the accuracy of this method, the qPCR detection protocol was compared with the PCR method established by Mabrok et al. (2020) [27]. Gill tissues were collected from six healthy grass carp and six with columnaris disease, and DNA (including gill tissues and bacteria) was extracted for both qPCR and PCR analysis. The qPCR results indicated Ct values between 17 and 23 for diseased grass carp and values exceeding 35 for healthy grass carp (Figure 11). These results confirmed that the qPCR method is consistent with the earlier PCR method.

### 3.4. Dynamic Changes in Bacteria Load Under Different Challenge Models

#### 3.4.1. Gills

**Figure 12:**
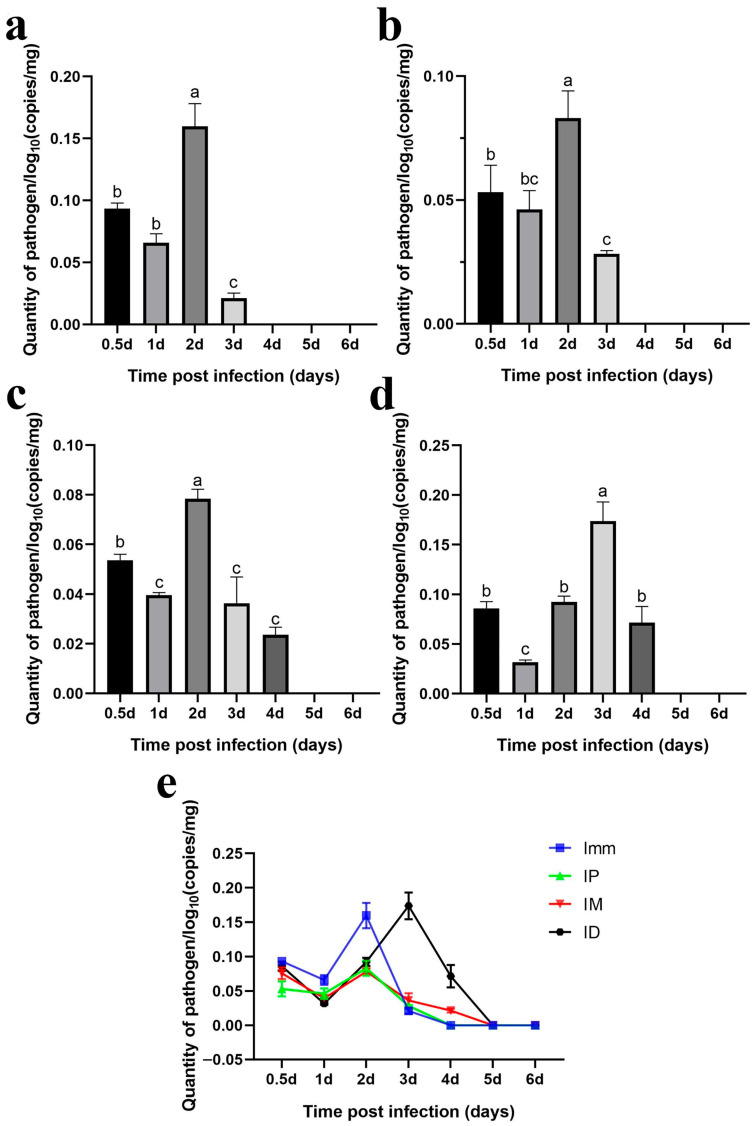
Dynamic distribution of bacterial loads in the gills of grass carp after challenge. (**a**) Dynamic distribution of bacterial loads after immersion bath; (**b**) dynamic distribution of bacterial loads after intradermal injection; (**c**) dynamic distribution of bacterial loads after intraperitoneal injection; (**d**) dynamic distribution of bacterial loads after intramuscular injection; (**e**) dynamic distribution of bacterial loads with the four different methods. A significance analysis was performed, and “a”, ”b”, ”c”, ”bc”, represented the significant differences.

Immersion challenge (Imm): At 0.5–1 days post-infection, *F. covae* loads in the gills showed a slight decrease, but it did not reach statistical significance. Bacterial loads then increased significantly between 1 and 2 days post-infection, before sharply decreasing at 3 days. By days 4, 5, and 6 post-infection, *F. covae* was undetectable in the gills (Figure 12a).

Intraperitoneal injection challenge (IP): Bacterial loads in the gills were highest on the second day post-infection, significantly decreasing by day 3 and becoming undetectable by day 4. The trend in bacterial load across other time points mirrored those of the immersion and intradermal injection groups (Figure 12b).

Intramuscular injection challenge (IM): Bacterial loads decreased slightly from 12 h to 1 day post-infection, followed by a significant increase. The peak load occurred on day 2, after which the bacterial levels gradually decreased, becoming undetectable in the gills by days 5 and 6 (Figure 12c).

Intradermal injection challenge (ID): From 12 h to 3 days post-infection, bacterial loads increased gradually, peaking at day 3, with the exception of a slight decrease on day 1. By day 4, the bacterial load dropped significantly, becoming undetectable by days 5 and 6 (Figure 12d).

Across all challenge groups, bacterial loads initially decreased by day 1 and then increased, peaking before gradually decreasing. The immersion and intradermal injection groups exhibited the highest bacterial loads throughout the infection period, with the immersion group showing the peak load on day 2. At this point, the bacterial levels in the other groups were comparable. However, the intradermal injection group reached its peak on day 3, with significantly higher bacterial loads than the other groups. Ultimately, bacterial loads in the gills of grass carp decreased across all groups, with *F. covae* becoming undetectable in the later stages of infection (Figure 12e).

#### 3.4.2. Skin (with a Small Amount of Muscle Tissue)

**Figure 13:**
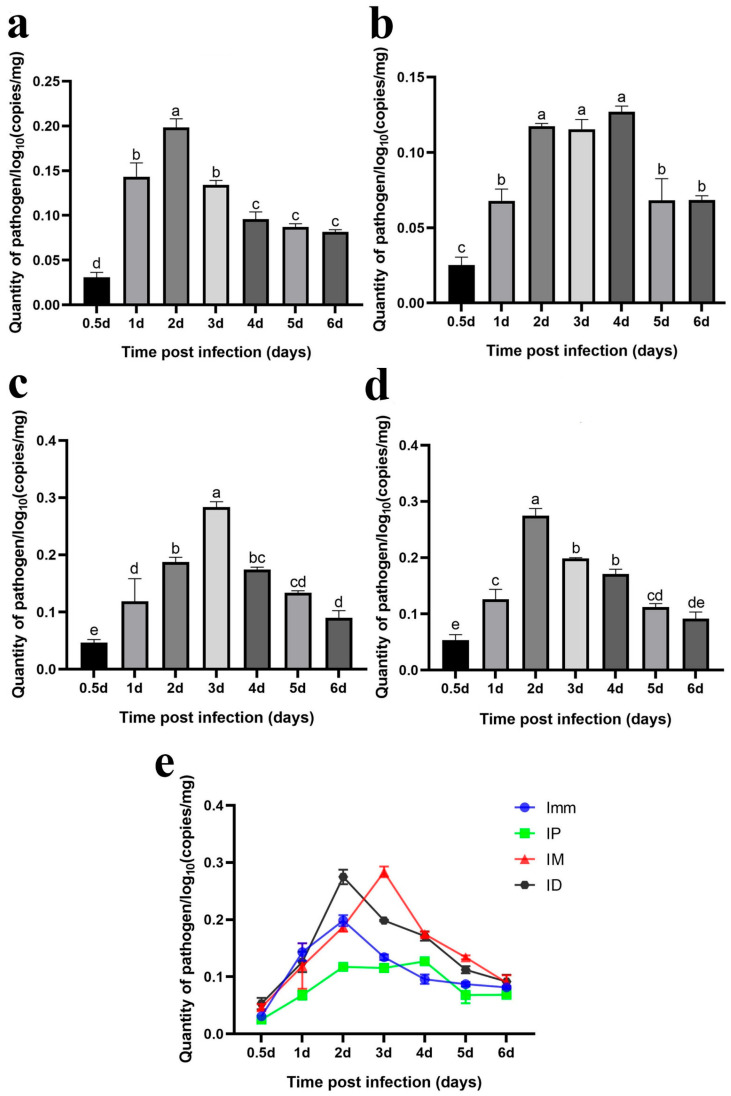
Dynamic distribution of bacterial loads in the skin (muscle) of grass carp after the challenge. Dynamic distribution of bacterial loads after (**a**) immersion bath; (**b**) intradermal injection; (**c**) intraperitoneal injection; (**d**) intramuscular injection. (**e**) Dynamic distribution of bacterial loads with the four different methods. A significance analysis was performed, and “a”, ”b”, ”c”, ”d”,”e”, “bc”, “cd”, “de” represented the significant differences.

Immersion challenge (Imm): Bacterial loads in the skin and muscle tissue of grass carp increased from 12 h to 2 days post-infection, peaking on day 2. Afterward, the bacterial levels gradually declined (Figure 13a).

Intraperitoneal injection challenge (IP): *F. covae* loads in the grass carp showed a significant increase from 12 h to 2 days post-infection, followed by a plateau over the next 2 days. By day 5, the bacterial load decreased significantly and remained stable at that level on day 6 (Figure 13b).

Intramuscular injection challenge (IM): Bacterial loads showed a significant increase within 12 h to 3 days post-infection, peaking on day 3. After day 3, bacterial loads declined significantly over time (Figure 13c).

Intradermal injection challenge (ID): The changes in *F. covae* loads in the skin (muscle) of the intradermal injection group mirrored those of the immersion group. Bacterial loads also reached their peak at 2 days post-infection (Figure 13d).

The *F. covae* bacterial loads in the skin (muscle) of grass carp were lower in the immersion and intraperitoneal injection groups compared to the other two groups, especially in the intraperitoneal group. Within 1 day post-infection, bacterial loads were similar in the immersion, intradermal, and intramuscular groups but differed in the intraperitoneal group. In the intraperitoneal group, the number of skin bacteria surged dramatically between 1 and 2 days post-infection, peaking on day 2. In contrast, bacterial loads in the immersion and intramuscular injection groups remained stable on day 2, but on day 3, bacterial loads in the immersion group plateaued, while they continued to rise in the intramuscular injection group, reaching a peak comparable to that in the intradermal injection group. Across these four infection groups, bacterial loads in the skin (muscle) initially increased and then decreased, but the bacterial load on day 6 exceeded that at 12 h (Figure 13e).

The previous two sections presented a specific qPCR method and reported temporal changes in detected bacterial content after infection. The results showed that the trends in bacterial loads in the gills and skin were similar, and bacterial loads in the intradermal injection group were higher than those in the other groups.

## 4. Discussion

*F. covae*, once thought to be *Flavobacterium columnare*, is now recognized as a distinct pathogen that causes columnaris disease in grass carp. Reliable and reproducible challenge models are essential to better understand this pathogen and its interactions with grass carp during infection, as well as to inform vaccine and treatment development. Effective challenge models are critical tools for studying pathogens and host interactions. A well-designed challenge model should not only induce a high mortality rate but also accurately replicate the natural disease symptoms. Many challenge methods suitable for higher animals are not applicable to fish due to the unique aquatic environment in which they live. Thus, challenge methods must be chosen based on the research objectives, the pathogen’s characteristics, the fish species, and the natural infection route [28].

Common fish infection methods include immersion and injection, each with its advantages and limitations regarding natural infection mimicry, bacterial dose control, and applicability to different fish species and life stages [29]. In this study, we assessed the pathogenicity of *F. covae* in grass carp using four challenge methods: immersion, intradermal injection, intraperitoneal injection, and intramuscular injection. The goal was to establish a stable challenge model that consistently replicates the typical clinical symptoms and high mortality rate of columnaris disease.

Given that fish naturally contact pathogens through their skin, gills, and digestive tract, the immersion challenge method mimics natural pathogen exposure in the aquatic environment [30,31,32]. Thus, we first validated the immersion challenge method based on prior studies [17,18,33]. Immersion with *F. covae* MU-04 caused a 70% mortality rate in 3–4 cm grass carp, a 15% mortality rate in 7–8 cm fish, and no mortality in larger grass carp. Since previous studies mainly used smaller fish for immersion challenges, subsequent experiments increased the bacterial dose and extended the challenge duration. When the bacterial loads reached 1.3 × 10^9^ cfu/mL and the exposure time was 4 h, 12 cm grass carp exhibited a 50% mortality rate. Moyer (2007) [17] demonstrated that in zebrafish, the LD50 of *F. columnare* strains ATCC 23,463 and Fc14-56 was >1 × 10^8^ cfu/mL when the skin was intact. However, when the skin was damaged, the LD50 for these strains dropped to 1.1 × 10^7^ and 1.1 × 10^6^ cfu/mL, respectively. Based on repeated experiments, the mortality rate for grass carp larger than 10 cm does not exceed 80%, regardless of the presence or absence of body surface damage. This suggests that fish size may significantly influence the results of immersion infection. Similar conclusions have been reached in previous studies [34].

The IP injection method is straightforward, rapid, and able to effectively deliver pathogens throughout the body [35]. In this study, grass carp challenged with *F. covae* MU-04 by IP injection had a mortality rate ranging from 73.4% to 86.7%, with an average symptom replication rate of approximately 25%, the lowest among the four methods tested. Figueiredo (2005) [21] reported a cumulative mortality rate of only 7% in channel catfish challenged with *F. columnare* through IP injection. In contrast, Su (2020) [36] found that an *F. columnare* infection dose of 1.25 × 10^8^ cfu/mL caused 11.11% mortality in adult rare minnows. Increasing the dose to 2.23 × 10^8^ cfu/mL resulted in 16.67% mortality, while 3.97 × 10^8^ cfu/mL caused 38.89% mortality. At 7.07 × 10^8^ cfu/mL, mortality reached 83.33%. However, no mortality occurred at doses of 3.94 × 10^7^ cfu/mL or 7.02 × 10^7^ cfu/mL, aligning with the findings from this study, where the highest dose of *F. covae* MU-04 (5 × 10^8^ cfu/mL) delivered through IP injection resulted in no death. These results suggest that IP injection may require a higher bacterial dose to induce mortality. Notably, there are no other reports on the intraperitoneal injection of *F. columnare* or *F. covae*, which further supports the hypothesis that IP injection may not be the optimal method for challenging these surface-dwelling bacteria.

Intramuscular injection is a commonly used technique to introduce pathogens directly into fish muscles. In this study, grass carp challenged with *F. covae* MU-04 through intramuscular injection exhibited mortality rates ranging from 26.7% to 100%, with symptom replication in deceased fish ranging from 25% to 75%. Previous studies using intramuscular injection of *F. columnare* to infect *Poecilia sphenops* showed mortality rates of 20–40% [2]. Barony (2015) [20] found that 10^6^ cfu/fish of *F. columnare* resulted in 100% mortality in Amazon catfish and 66.7% in pacamã. Similarly, Figueiredo et al. (2005) [21] reported 80% mortality in channel catfish and 35% in tilapia following intramuscular injection of 10^6^ cfu/fish. Welker (2005) [18] injected 1.25 × 10^8^ cfu/mL of *F. columnare* into channel catfish, resulting in 90% mortality. The results from this study, combined with prior research, indicate that the mortality rate from intramuscular injection is influenced by factors such as fish species, size, and strain virulence.

No studies were found regarding intradermal injection as a challenge model in fish, although this method is more commonly used in mammalian models. *F. columnare* accumulates between collagen fibers in the dermis of fish following natural infection, prompting its inclusion in this study [37,38]. Our experimental results revealed that, at the same challenge dose as the other three methods, intradermal injection led to an average symptom occurrence of approximately 50%. In the medium- and low-dose challenge groups, skin symptoms occurred in over 80% of the fish, likely due to the injection method and site. The typical gill symptoms appeared in over 50% of the fish, with 100% mortality in both the medium- and high-dose intradermal injection groups. Compared with other injection methods, the intradermal group exhibited a higher probability of showing typical symptoms of columnaris disease. In contrast, the intradermal injection group demonstrated higher mortality and a more consistent pattern than the immersion challenge group. However, the current study has some limitations, including the reliance on a single bacterial strain and fish species, as well as the omission of environmental factors such as temperature and water quality that may influence grass carp. Further research is still needed to address these issues.

The clinical symptoms of grass carp columnaris disease, simulated through four different challenge methods in this study, varied significantly. After intraperitoneal injection, no obvious gill or skin symptoms appeared in the grass carp, and the replication of typical columnaris disease symptoms was low. This may be due to the pathogen’s primary infection of the body surface under natural conditions, making intraperitoneal injection less effective. In contrast, intramuscular injection resulted in clear ulceration of the back skin and muscles, with severe yellow erosion of the gills. The replication of typical symptoms was higher in dead fish, indicating a stronger disease response. After intradermal injection, grass carp developed distinct “saddleback” lesions on their dorsal fins, with visible yellow biofilms around the affected areas. Additionally, the gills were filled with sticky yellow substances. The symptom replication rate was high, similar to that in the intramuscular injection group, but skin ulceration was more severe following intramuscular injection, which could cause greater damage to the fish. Used as a control, immersion infection caused a high proportion of dead fish to exhibit gill and skin symptoms. While this method simulated the natural disease state, it did not ensure a stable mortality rate, showing that immersion infection is not sufficiently reliable for consistent disease modeling.

Among the four challenge methods evaluated in this study, the primary histopathological alterations observed in grass carp gills were lamellar hyperplasia and fusion. The most severe symptom occurred at 3 dpi in the immersion and intradermal injection groups, whereas it peaked at 3 dpi in the intramuscular and intraperitoneal injection groups, which is consistent with previous results [39,40]. In the immersion challenge group, the gills were directly exposed to the pathogen, resulting in rapid transmission. For intradermal injection, the extensive vascular network in the dermal layer likely facilitated bacterial entry into the bloodstream, enabling quick infection of the gills. Previous research has demonstrated that infection induces characteristic dermatopathological manifestations, including epidermal edema, necrosis, and scale detachment. The infection progressively extends into subcutaneous tissues, accompanied by capillary rupture and hemorrhage [3,38]. In the present study, all three challenge methods (immersion, intradermal injection, and intramuscular injection) elicited comparable skin pathological alterations in grass carp.

To verify that the pathological symptoms were caused by artificial infection, we developed a qPCR assay and subsequently employed this method to quantify bacterial load dynamics in grass carp tissues across the different infection model groups. The results showed that, in the gill tissue, bacterial counts of *F. covae* MU-04 were higher in the immersion and intradermal injection groups than in the other two groups throughout the infection process. The bacterial load trends in these two groups were nearly identical, but the peak bacterial load in the intradermal injection group occurred 1 day later than that in the immersion group. This delay likely reflects differences in pathogen exposure and transmission routes: intradermal injection likely allows the pathogen to reach the gills through the blood circulation, with proliferation occurring 1–2 days post-infection, while the immersion group is directly exposed to the pathogen, leading to an earlier peak [39,40].

Regarding bacterial load changes in skin/muscle tissue, within 1 day of infection, bacterial counts in all groups were similar, except in the intraperitoneal injection group. In the intradermal injection group, bacterial loads in the skin/muscle tissue increased rapidly within 1–2 days, while in the intramuscular injection group, they increased sharply on days 2–3. However, bacterial loads in the skin/muscle of the immersion group did not increase after 2 days, and the bacterial accumulation was lower than that in the intradermal and intramuscular injection groups. This discrepancy likely results from the lower infection load in the immersion challenge group.

In previous studies, Zhang and Gibbs established a qPCR method for detecting bacterial loads in tissues based on the chondroitin AC lyase gene of *Flavobacterium columnare*. They found an increase in bacterial loads in the gills, liver, spleen, and kidneys of channel catfish following infection [41,42]. Bader (2003) [19] also utilized PCR to detect *F. columnare* in the blood, gills, fins, mucus, liver, and kidneys of channel catfish after infection. Similarly, Decostere (1998) [4] reisolated *F. columnare* from the gills, mucus, and spleen of black mollies after artificial infection. Evenhuis (2014) [34] reported a 10% probability of reisolating *F. columnare* from the spleen and head kidney of rainbow trout infected with strain CSF289-10, while Barony (2015) [20] found *F. columnare* in the liver, kidneys, and spleen of *Piaractus mesopotamicus* after challenge. Additional studies have isolated *F. columnare* from the liver and kidneys of various fish species [27,43,44]. In contrast, our study showed that after challenging grass carp with *F. covae* MU-04, bacterial loads were detectable only in the gills and skin, while they were below the qPCR detection threshold in the liver, spleen, and kidneys. This may be due to the species diversity of *F. columnare* and its varying infection targets. The MU-04 strain, isolated from the surface-ulcerated muscles of diseased fish, may not invade visceral tissues. Furthermore, Decostere (1998) [4] observed that *F. columnare* was only randomly detected in the spleens of black mollies challenged with different doses of the same strain, suggesting that internal organ invasion by *F. columnare* may be an incidental event influenced by multiple factors. However, there is no doubt that the bacterium preferentially targets the gills and skin.

## 5. Conclusions

This study compared mortality rates, replication rates of clinical columnaris disease symptoms, pathological alterations, and bacterial loads in various tissues after *F. covae* challenge via immersion, intraperitoneal injection, intramuscular injection, and intradermal injection. Intradermal injection of *F. covae* into grass carp resulted in high mortality, a high probability of replicating typical columnaris symptoms, and significant bacterial loads in tissues. This reproducible challenge model most closely resembles the natural infection state. Based on these results, intradermal injection is considered the most effective infection route for *F. covae*, which can provide technical support for evaluating the protective effect of vaccines. Future studies with other fish or bacterial strains could also attempt to use this model for challenge and incorporate more environmental factors into experimental designs.

## Figures and Tables

**Table 1:** Replication of typical symptoms of columnaris disease with different challenge methods.

Challenge Method	Bacteria (cfu/Fish)	Mortality Rate	Typical Symptoms of Columnaris Disease % (Symptom Fish/Total Dead Fish)	
			Gill	Skin/Muscle
Immersion	1.3 × 10^10^	46.7%	43% (3/7)	43% (3/7)
	7 × 10^9^	53.3%	87% (7/8)	63% (5/8)
	1.3 × 10^9^	0	0%	0%
Intraperitoneal injection	5 × 10^8^	86.7%	15% (2/13)	23% (3/13)
	2.6 × 10^8^	73.4%	45% (5/11)	18% (2/11)
	5 × 10^7^	73.4%	18% (2/11)	18% (2/11)
Intramuscular injection	5 × 10^8^	100%	67% (10/15)	33% (5/15)
	2.6 × 10^8^	100%	53% (8/15)	40% (6/15)
	5 × 10^7^	26.7%	25% (1/4)	75% (3/4)
Intradermal injection	5 × 10^8^	100%	47% (7/15)	53% (8/15)
	2.6 × 10^8^	100%	53% (8/15)	91% (14/15)
	5 × 10^7^	40%	50% (3/6)	83% (5/6)

**Table 2:** Ct values of qPCR detection.

Strain	Reporter	CT
*Chryseobacterium* sp.	SYBR	Undetermined
*Flavobacterium indicum*	SYBR	Undetermined
*Novosphingobium panipatense*	SYBR	Undetermined
*Aeromonas veronii*	SYBR	Undetermined
*Stenotrophomonas* sp.	SYBR	Undetermined
*Aeromonas hydrophila*	SYBR	Undetermined
*Flavobacterium colunmare* JX-01	SYBR	30.687
*Flavobacterium covae* MU-04	SYBR	17.189

## Data Availability

The data presented in this study are available on request from the corresponding author due to privacy restrictions.
